# Mobile ions determine the luminescence yield of perovskite light-emitting diodes under pulsed operation

**DOI:** 10.1038/s41467-021-25016-5

**Published:** 2021-08-12

**Authors:** Naresh Kumar Kumawat, Wolfgang Tress, Feng Gao

**Affiliations:** 1 Department of Physics, Chemistry and Biology (IFM), Linköping University, 58183 Linköping, Sweden; 2 Institute of Computational Physics, Zurich University of Applied Sciences, Wildbachstr. 21, 8401 Winterthur, Switzerland

**Keywords:** Electronic devices, Applied physics

## Abstract

The external quantum efficiency of perovskite light-emitting diodes (PeLEDs) has advanced quickly during the past few years. However, under pulsed operation, an operation mode which is important for display and visible light communication, the performance of PeLEDs changes a lot and requires in-depth understanding to facilitate these applications. Here, we report the response of PeLEDs under pulsed operation in the range of 10 Hz to 20 kHz. Beyond transient effects in the low frequencies, we find that for higher frequencies (>500 Hz) the transient electroluminescence intensity depends strongly on the duty cycle. This feature is much more pronounced and of different origin than that in conventional LEDs. We rationalise our experimental observations using a mathematical model and assign these features to the effect of mobile ionic charges in the perovskite. Our work also provides important implications for the operation of PeLEDs under the steady state, where accumulation of mobile ions at the interfaces could be beneficial for high electroluminescence yields but harmful for the long-term stability.

## Abstract (web summary)

Pulsed operation of perovskite light-emitting diodes is of particular importance in display and visible light communication, yet the ionic behaviour under this mode is not well-understood. Here, the authors reveal that the transient electroluminescence intensity increases with increasing pulse width as the result of accumulation of mobile ions at the interfaces.

## Introduction

Perovskite light-emitting diodes (PeLEDs) have received increasing attention during the last few years due to their appealing properties such as high colour purity with wide colour gamut, easily tunable electroluminescence (EL) emission from the near-infrared (NIR) to the visible region, solution processability, fast-response, and mechanical flexibility^1–6^. In the last five years, most of the efforts in the field have focused on achieving high external quantum efficiency (EQE) and stable device operation. By employing compositional engineering, molecule additives, and device engineering strategies, EQEs of PeLEDs have reached high values over 20%, making them promising for cost-effective lighting and display applications^7–12^.

Despite these advances, a detailed understanding of how the ionic conductivity affects the operation of PeLEDs is currently missing, although pulse driving operations have been used to suppress the drawbacks of ionic motion and to improve the device stability of PeLEDs^13–20^. Ionic transport is an intrinsic property of perovskites, with slow response times in the time scale of millisecond (ms) to second^21^. In perovskite solar cells, ionic transport has been analysed in great detail and identified as origin of the hysteresis in the current–voltage curve and degradation of the perovskite, charge transport layers and the metal electrodes^22–25^. Various strategies have been applied to alleviate the negative effects of mobile ions including molecular additives, metal doping, and interface engineering. In PeLEDs, slow response of ions becomes relevant under pulsed operation, e.g., for lighting and displays, visible light communication (VLC), and lasers. Therefore, investigations are required to deeply understand the behaviour of PeLEDs under pulsed operation, which is important for practical applications of this emerging technology.

Here, we investigate the transient electroluminescence (TrEL) of highly efficient NIR PeLEDs under pulsed operation in the range of 10 Hz to 20 kHz. At low frequencies, we observe the expected transients caused by ionic movement in the TrEL and the transient current density (Tr. J). However, for higher frequencies, where the mobile ions are not supposed to respond, the TrEL intensity remains a function of the duty cycle. Investigating various device architectures and perovskite materials, we find that this phenomenon, despite being related to the working principle of the devices, is a common property of PeLEDs and not specific to a certain device architecture or perovskite composition. We present a mathematical model that reproduces the varying TrEL intensity and find that the underlying mechanism at high frequencies can also be explained by ionic movement with a time constant of 10 ms, consistent with the dynamics of mobile ionic charges in perovskites.

## Results and discussion

### Device performance and stability of NIR PeLEDs

**Fig. 1:**
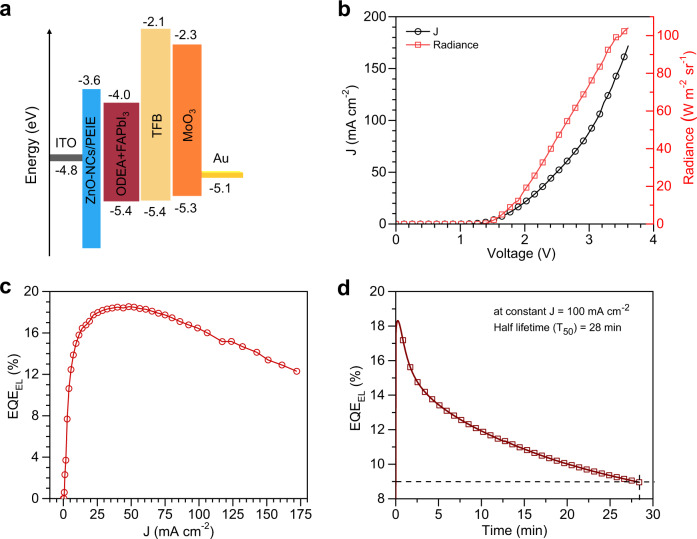
Device performance and stability characterisations of the near-infrared (NIR) perovskite light-emitting diodes (PeLED). **a** Energy levels of the materials used in the NIR PeLED. **b** Current density-voltage-radiance (J–V–R) characterisation of the champion device, maximum radiance of 104 W m^−2^ sr^−1^ at 3.6 V. **c** Electroluminescence external quantum efficiency (EQE_EL_) versus current density curve, peak EQE_EL_ of 18.5% at 50 mA cm^−2^. **d** Device stability curve at a constant current density of 100 mA cm^−2^ to obtain half lifetime (*T*_50_ = 28 min) of the PeLED.

Figure 1a shows the energy levels of the materials used in the NIR PeLED consisting of indium tin oxide (ITO)/zinc oxide nanocrystals (ZnO-NCs)/polyethylenimine ethoxylated (PEIE)/formamidinium lead tri-iodide (FAPbI_3_) perovskite with ODEA (2,2-(oxybis(ethylenoxy)) diethylamine)) additive/poly(9,9-dioctyl-fluorene-co-N-(4-butylphenyl)diphenylamine (TFB)/molybdenum oxide (MoO_3_)/Au device structure. ODEA works as a defect passivation agent that improves the performance and stability of the NIR PeLED^10^. EL spectra of the NIR PeLED peak at 805 nm with full width at half maximum (FWHM) of 59 nm. The shape of the EL spectra does not change with an increasing bias, which is shown in Supplementary Fig. 1. Figures 1b, c show current density–voltage–radiance (J–V–R) and electroluminescence external quantum efficiency (EQE_EL_)-J characteristics of the NIR PeLED, respectively. The PeLED shows maximum radiance of 104 W m^−2^ sr^−1^ at 3.6 V and an EQE_EL_ of 18.5% at 50 mA cm^−2^. We performed stability measurement on this device at a constant current density of 100 mA cm^−2^, and obtained a half lifetime (T_50_) of 28 min (Fig. 1d). EQE_EL_ and stability data of the NIR PeLED suggest that the device is efficient and sufficiently stable to investigate its pulsed operation.

### Pulsed operation of the NIR PeLEDs

**Fig. 2:**
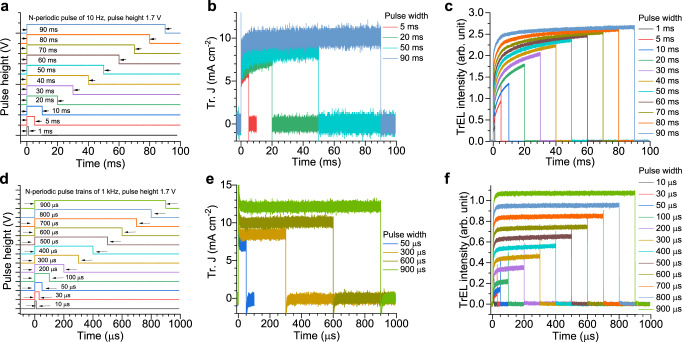
Pulse width dependent transient electroluminescence (TrEL) characterisations of the near-infrared (NIR) perovskite light-emitting diode (PeLED) at 10 Hz and 1 kHz. **a** Schematic diagram of *N-*periodic pulse trains of 10 Hz frequency, wherein black arrows show voltage pulse widths (from 1 ms to 90 ms), which are used to excite the PeLED. **b** Transient current density (Tr. J) and **c** TrEL intensity as a function of the pulse widths, which are shown in Fig. 2a. **d** Schematic diagram of *N-*periodic pulse trains of 1 kHz frequency, wherein black arrows show voltage pulse widths (from 10 μs to 900 µs), which are used to excite the PeLED. **e** Tr. J and **f** TrEL intensity as a function of the pulse widths, which are shown in Fig. 2d. For the figure clarity, here, we show Tr. J only for selected pulse widths; for the other pulse widths at 10 Hz and 1 kHz, Tr. J curves are shown in Supplementary Figs. 3b and  8b, respectively. TrEL and Tr. J data have also been measured for the other pulse widths in the range of 10 Hz to 20 kHz (Supplementary Figs. 3 to  11). Both Tr. J and TrEL intensity increase with increasing pulse widths for all the frequencies we investigated.

The transient current density (Tr. J) and the TrEL are recorded for various frequencies (10 Hz to 20 kHz) and pulse widths (Supplementary Figs. 3 to 11) using a setup, which is shown in Supplementary Fig. 2. To achieve reproducible results, all measurements are performed inside a nitrogen filled glovebox and representative high-frequency and low-frequency results are displayed in Fig. 2. Figure 2a, d show a schematic diagram of rectangular periodic electrical pulse widths (pulse height is 1.7 V) of 10 Hz and 1 kHz frequency, respectively that are applied to the PeLED. Figure 2b, e show the Tr. J as a function of pulse widths for 10 Hz and 1 kHz, respectively. The Tr. J curves (Fig. 2b,e) show sharp overshoots when the voltage is switched, which are attributed to capacitive charging and discharging of the device^26^. The saturated transient current density (Tr. J_S_) signal originates from injection current into the device. The Tr. J at 10 Hz shows a response time in the millisecond regime (Supplementary Fig. 12) and overall decreasing values with a decreasing pulse width (Fig. 2b). For the 1 kHz data (Fig. 2e), the transients cannot be seen but the trend of a reduced Tr. J for lower pulse widths remains. Variations of the Tr. J_S_ are relatively constant at 40% between the small and large pulse widths in all the frequencies (Supplementary Fig. 13). This suggests that charge transport or injection might be improved for longer pulses independent of driving frequency. The transients seen at 10 Hz, are observed up to 200 Hz (Tr. J data, Supplementary Figs. 3 to  6), and supposed to reflect the response time of mobile ions^27^.

In contrast to the Tr. J, the TrEL shows a much more pronounced dependence on the pulse width as seen in Fig. 2c, f, and Supplementary Figs. 3 to  11. Particularly interesting is the behaviour at higher frequencies (Fig. 2f), where the turn-on (<50 µs) is followed by a rather constant TrEL, whose value significantly decreases with reduced pulse width. Such a phenomenon of that significance has never been observed in any traditional LEDs^28–30^. To make sure that the TrEL signal does not depend on the history of the device, we also recorded it in various sequences of the pulse width (e.g., forward, backward, and random). Supplementary Fig. 14 to Fig. 17 show that the TrEL is independent of the sequence. Furthermore, J-V-R measurements, performed before and after the TrEL measurements (Supplementary Fig. 18), show that the device hardly degrades during the TrEL measurements. In addition to the different plateau values in TrEL, giant overshoots are observed at the end of the pulses, where the intensities of the overshoots decrease with an increasing pulse width. Similar overshoots were observed in perovskite and other LEDs and attributed to the recombination of electrons and holes that accumulated at the interfaces with the charge transport layers^27,31–33^.

In addition, we investigate how the pulsed operation affects the device operational stability (Supplementary Figs. 19 and 20). The device lifetime increases with increasing frequencies (at a fixed duty cycle, Supplementary Fig. 19), possibly because alternating ionic movement is alleviated at high frequencies where ions can hardly respond. The device lifetime also increases with increasing duty cycles (at fixed frequencies, Supplementary Fig. 20). Since the EL intensity increases with increasing duty cycles, larger duty cycles require smaller driving voltages to reach a constant EL output under which the lifetime is measured. Smaller driving voltages for larger duty cycles benefit the device lifetime. Thus, pulsed operation at the same average output EL intensity seems not a way to increase device stability.

### Independence of the device architecture

To understand whether the enhanced Tr. J and TrEL intensity with an increasing pulse width is specific to NIR PeLEDs, we further investigate other PeLEDs with different device architectures and materials. We choose the quasi-two-dimensional (2D) PeLED with ITO/poly(3,4-ethylenedioxythiophene polystyrene sulfonate(PEDOT:PSS)/TFB/poly(9-vinlycarbazole)(PVK)/quasi-2D perovskite/2,2′,2”-(1,3,5-Benzinetriyl)-tris(1-phenyl-1-H-benzimidazole)(TPBi)/lithium fluoride (LiF)/Al device architecture  (Supplementary Fig. 21a), which is completely different as compared to NIR PeLEDs (Fig. 1a). This device shows a peak EQE_EL_ of 7.2%, luminance of 922 cd m^−2^, and EL emission peak wavelength at 650 nm (Supplementary Fig. 21b, c). From the TrEL measurement performed on this device, we find that the Tr. J and TrEL intensity increase with an increasing pulse width (Supplementary Fig. 21d–f), similar to what we have seen for the NIR PeLED (Fig. 2). In addition, for quasi-2D PeLED we also observe the overshoot, the intensity of which decreases with increasing pulse widths. Now we can conclude that enhanced Tr. J and TrEL intensity of PeLEDs with an increasing pulse width are a common property in various PeLEDs and not specific to a certain device architecture. In contrast, similar measurements on other LEDs (like organic LEDs, Supplementary Fig. 22a, b) show significantly different results, with weak dependence of the TrEL intensity on the pulse widths and weak overshoot (Supplementary Fig. 22c, d), consistent with previous reports^28–30^.

### The importance of the duty cycle

**Fig. 3:**
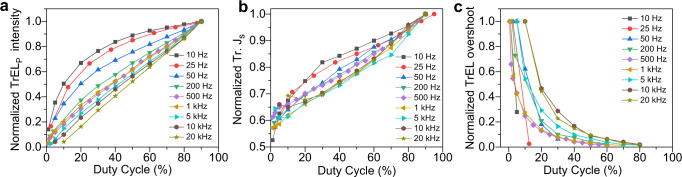
Transient electroluminescence (TrEL) dynamics versus the duty cycle as a function of driving frequency. **a** Normalised TrEL_P_ intensity versus the duty cycle. For this figure, we use plateau values (TrEL_P_ intensity values at the end of the TrEL pulse) from the TrEL data, which are recorded for different pulse widths in the range of 10 Hz to 20 kHz (Supplementary Figs. 3 to  11). **b** Normalised saturated transient current density (Tr. J_S_) versus the duty cycle. **c** Normalised TrEL overshoot intensity versus the duty cycle.

In order to gain deep insights and quantify this effect, we now focus on its dependence on frequency and duty cycle $({{D}}\,( \% )=\frac{{{{t}}}_{{{{{{\rm{on}}}}}}-{{{{{\rm{time}}}}}}\,{{{{{\rm{pulse}}}}}}\,{{{{{\rm{width}}}}}}}}{{{{t}}}_{{{{{{\rm{off}}}}}}-{{{{{\rm{time}}}}}}\,{{{{{\rm{pulse}}}}}}\,{{{{{\rm{width}}}}}}}\,+\,{{{t}}}_{{{{{{\rm{on}}}}}}-{{{{{\rm{time}}}}}}\,{{{{{\rm{pulse}}}}}}\,{{{{{\rm{width}}}}}}}}\times 100)$. Figure 3a shows the TrEL intensity (TrEL_P_) (normalised at *D* = 90%) at the end of the TrEL pulse in a range of 10 Hz to 20 kHz. For larger frequencies (>500 Hz), the TrEL_P_ is a linear function of *D*, independent of frequency. In contrast, towards lower frequencies, the TrEL_P_ loses its linearity with the duty cycle as the slow process is resolved. We also change the sequence of the measurements and observe similar results (Supplementary Fig. 17), indicating that the dependence on the duty cycle is not affected by the sequence of the experiments.

The saturated transient current density (Tr. J_S_) at the end of the pulse (Fig. 3b) shows a similar behaviour, however, with a much weaker relative change of the Tr. J_S_ with *D*. This result indicates that the TrEL_P_ in Fig. 3a is a better parameter to resolve the slow process compared with the current density in Fig. 3b. As we observe an increased overshoot with reduced pulse width in Fig. 2, we also investigate whether the absolute pulse width or *D* dominates the overshoot. The normalised TrEL overshoot intensities in Fig. 3c confirm that *D* is the decisive parameter. The decrease of overshoot intensity with *D* accompanying the increase of TrEL_P_ with *D* might have their common origin in mobile ionic charges^19^ as will be discussed in the next section.

### Mathematical model

The various experiments have shown that the plateau value of the TrEL signal (TrEL_P_) depends mainly on the duty cycle and not on the absolute value of on-time, off-time, or frequency for sufficiently large frequencies. Whereas the TrEL scales approximately linearly with the duty cycle, leading to ten times increase of the TrEL when comparing 10% with 90% duty cycle (Fig. 3a), the Tr. J_S_ changes only by around 1.6 times (Fig. 3b). Therefore, the main change is in the EL yield. Interestingly, the overshoot after turning off the voltage shows an inverse trend with the duty cycle (Fig. 3c).

All these observations can be explained with the following model: We assume that the TrEL intensity is influenced by a slow process that cannot be resolved in a measurement in the kHz regime, rationalising the frequency dependence for low frequencies in our measurements. The duty cycle dependence at high frequencies also results from the slow process because the accumulated effect of the slow processes becomes visible in the TrEL in high frequencies as described in the following.

We describe the slow process by introducing a probability function *P*, which rises during the on-time

1 $${{{P}}}_{{{{{{\rm{rise}}}}}}}({{t}})={{{P}}}_{{{{\rm{off}}}}}+({{{P}}}_{{{{{{\rm{max }}}}}}}-{{{P}}}_{{{{{{\rm{off}}}}}}})\left(1-\exp \left(-\frac{{{t}}}{{{\tau }}}\right)\,\right);\,{{t}}=[0,\,{{{t}}}_{{{{{{\rm{on}}}}}}}]$$

and decreases during the off-time;

2 $$\,{{{P}}}_{{{{{{\rm{decay}}}}}}}({{{{t}}}})={{{P}}}_{{{{{{\rm{on}}}}}}}\exp \left(-\frac{{{t}}-{{{t}}}_{{{{{{\rm{on}}}}}}}}{{{\tau }}}\right);\,{{t}}=\left[{{{t}}}_{{{{{{\rm{on}}}}}}},\frac{1}{{{f}}}\right]$$

**Fig. 4:**
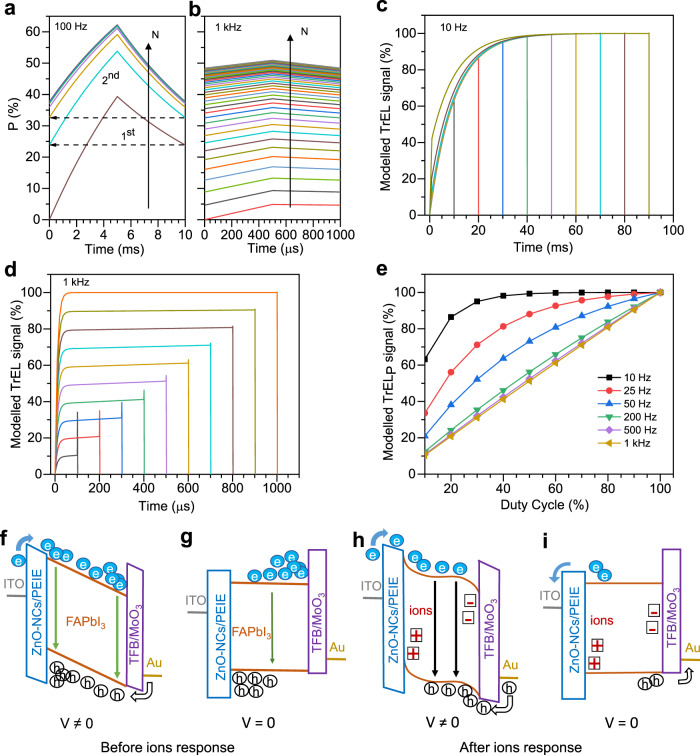
Modelled transient electroluminescence (TrEL) dynamics as a functions of pulse width and frequency. Probability function P as defined in the Eqs. (1) and (2) for **a** 100 Hz and **b** 1 kHz. The arrow (solid vertical black) indicates the increasing cycle number *N*. For the 1st and 2nd cycle, the dashed arrows indicate that the start value of a new cycle is the end value of the preceding cycle. **c** and **d** Modelled converged TrEL signal as a function of pulse width for 10 Hz and 1 kHz, respectively. **e** Modelled TrEL_P_ signal versus duty cycle as a function of frequency variation**. f**–**i** Sketch of the band diagram after applying a positive forward voltage pulse **f**, **g** before and **h**, **i** after ions responded. The vertical arrows indicate regions of high recombination.

These functions approach their final value (*P*_max_ and 0) with an exponentially decaying difference described by the time constant *τ*. The time *t* is reset for each cycle, i.e., *t* = *t*_overall_ − *N*/*f*, where *N* is a natural number describing the cycle and *f* is the frequency. The starting values *P*_off_ and *P*_on_ are the final values of preceeding ${{{P}}}_{{{{{{\rm{decay}}}}}}}(\frac{1}{{{f}}},{{N}}-1)$ and ${{{P}}}_{{{{{{\rm{rise}}}}}}}({{{t}}}_{{{{{{\rm{on}}}}}}},\,{{N}})$, respectively. The choice of such a function is motivated by the current and TrEL response at low frequencies, where an exponential behaviour with *τ* ≈ 10 ms can be deduced (Supplementary Fig. 12). When performing pulsed operation at high frequencies, this transient response is not directly observable. However, Fig. 4a, b shows that it has effect on the TrEL signal. Figure 4a shows how *P* evolves after turning on a pulsed signal with duty cycle of 50% at 100 Hz. The exponential feature starting at 0 can be well observed in the first cycle. Before reaching its maximum value, the voltage is switched off and the slow process decays with the same time constant. However, before it fully decays, the next voltage pulse arrives and the signal does not start from 0. This procedure continues and after several cycles the signal converges and describes a quasi-steady-state situation. The number of cylces needed for reaching the quasi-steady-state situation scales with *f*τ. Figure 4b shows the same simulation for 1 kHz.

In the experiment, directly the converged signal is measured, because the signal is recorded and averaged with an oscilloscope during a time of seconds. Therefore, we take the converged transient and calculate it for different on-times of the pulse. We assume that the TrEL is proportional to *P*_rise_, when the voltage is on, whereas *P*_decay_ happens invisibly as there is no TrEL when the driving voltage is off. To account for the electronic onset of the TrEL with a time constant of around 10 µs (visible in Fig. 2f), we add a process with this short rise time to the model. We, furthermore, inculde a phenomenological overshoot that decreases exponentially with the TrEL signal before switching off, which is justified by the experimental observations shown in Fig. 3c. The obtaind results are plotted in Fig. 4c, d for 10 Hz and 1 kHz, respectively. They show very good coincidence with the experimental data (Fig. 2c, f). For the low frequency (Fig. 4c), the response of the slow process can be well resolved, whereas for the high frequency (Fig. 4d), the plateau value scales with the on-time and only for lower on-times a small slope shows the effect of the slow process. This effect on the slope is also clearly visible in the experiment.

As a next step we vary the frequency and plot the modelled TrEL_P_ signal (end value during on time) versus duty cycle as in Fig. 3a. We observe that for frequency ≥200 Hz, the TrEL_﻿P_ becomes independent of frequency (Fig. 4e). This is expected as long as ${{{{{\rm{\tau }}}}}}\gg \,1/{{{{f}}}}$, because quasi-steady-state can be reached and the result just depends on the ratio of on and off-time (or duty cycle). For lower frequency, the slow process can be more and more resolved, which ultimately (here for 10 Hz) results in a TrEL following Eq. (1) with *P*_off_ = 0 for each cycle. Also this data is perfectly consistent with the experiment.

Whereas we have discussed a purely mathematical model so far, we now explain the underlying physics with Fig. 4f–i. After applying a forward voltage, a positive electric field is generated within the perovskite, which drives electron and hole transport. As these charges move fast and might be injected in an imbalanced way, they accumulate at the blocking electrode, where they recombine (Fig. 4f). This situation causes mainly non-radiative recombination due to spatial separation of charges and recombination predominantly at surfaces through surface/interface defects. Therefore, the EL yield is relatively low. When turning off the voltage, charges diffuse towards the centre, where radiative recombination is more likely (Fig. 4g). This is not visible in the external current but results in an overshoot in the TrEL, as Gegevicius et al. showed^27^.

If the voltage remains on for longer, mobile ions in the perovskite have time to respond and tend to accumulate at the interfaces screening the electric field and causing a situation as depicted in Fig. 4h^19^, analogous to what has been discussed in perovskite solar cells^22^. This situation facilitates charge injection at the electrodes and the reduced field in the bulk enhances recombination of charges there. Thus, the overall TrEL intensity increases and at the same time the overshoot vanishes (Fig. 4i). Measuring EL spectra as a function of duty cylce (Supplementry Fig. 1a) we could not observe any changes in the spectra, which could have been a hint for shifting recombination zones. Thus, consistent with the overshoot, the major radiative recombination zone remains at the same position and the recombination close to the interfaces is mainly non-radiative.

In the pulsed mode at higher frequencies, the ion distribution yields a result inbetween these two extreme situations, with an effect linearly depending on the duty cycle as the simulation shows. Therefore, the TrEL plateau scales with the duty cycle and the lower the duty cycle, the lower the TrEL and the higher the overshoot. The experimental data in Fig. 3c confirms this effect on the overshoot, which does not depend on absolute pulse lenghts but mainly on the duty cycle. The overshoot never exceeds the plateau emission for high duty cycles, meaning that previous ideas of obtaining extraordinarily high emission yields in the overshoot are not confirmed^27^. The time scale of 10 ms is consistent to reports on ion migration in perovskite solar cells^34,35^. In the simulation, we can employ different values of τ for *P*_rise_ and *P*_decay_. As Supplementry Fig. 23 shows, this does not lead to a linear scaling, which indicates that in experiment ion accumulation and reequilibration happen on the same time scale. However, the occurrence of a faster rise with enhanced duty cycle in the experimental data for small frequencies (Fig. 2c), which is absent in the simulation data for small frequencies (Fig. 4c), might be explained with a slightly higher τ for the decay (Supplementry Fig. 24).

The dependence of the EL intensity on the preceding share of on-time constitutes a memory-feature that might become interesting for novel computing based on simultaneous information processing and storage in one device. The simple two-terminal LED integrates the input signal or, in case of pulsed operation, converts information on the duty cycle of previous pulses into a proportional emission signal. Whereas this effect needs to be additionally taken into consideration when designing drivers for displays with PeLEDs, such a kind of LED with memory effect might become interesting for future electronics with memristive elements^36–40^.

In addition, the mechanisms revealed from the pulse operation also have important implications for the operation of PeLEDs at the steady state. The accumulation of ions at the interfaces, which can relax during the off mode in the pulse operation, might penetrate into the charge-transport layer in the steady state, being detrimental to the device stability. For future development of highly efficient and stable PeLEDs, further efforts might be required to improve the balance between electrons and holes, e.g., through optmising the charge transport layers. As long as balanced charge injection is reached, crystal engineering of perovskite can help to minimise the ionic movement, as demonstrated in perovskite solar cells^41–45^. Optimisation of charge transport layers requires optimal energy level alignment and at the same time sufficient conductivity and blocking behaviour to reach high selectivity. Here, effective doping of the charge transport layers might be an approach to instantaneously reach high luminescence yields without the need for ion displacement.

In summary, we have investigated the TrEL behaviour of PeLEDs under pulse mode operation with varying periodic electric pulse widths (or duty cycle), and repetition rate. We have shown that the TrEL intensity increases with increasing pulse width, where the duty cycle is the determining factor for this interesting behaviour. These phenomena are found in both 3D and quasi-2D PeLEDs with different device structures, suggesting that the phenomena originate from the perovskite materials and occur in various device structures. Our mathematical model explained that the change in TrEL intensity is due to the slow process of mobile ions. These mobile ions change the internal electric field, which leads to a modified share of radiative recombination and hence change in TrEL intensity. This study demonstrates a unique transient behaviour of PeLEDs, paving ways for further development of perovskite-based lighting and display, segment displays, and VLC applications. In addition, it provides new understanding especially when the PeLEDs are used in the pulsed operation, and also has important implications for the operation of PeLEDs under steady state.

## Methods

### Chemicals

2,2′-(oxybis(ethylenoxy) diethylamine (ODEA), 2-phenoxyethylamine (POEA), poly (9-vinlycarbazole) (PVK), lithium fluoride (LiF), polyethylenimine ethoxylated (PEIE), caesium iodide (CsI) and dimethylformamide (DMF) were purchased from Sigma-Aldrich. Formamidinium iodide (FAI) and methylammonium iodide (MAI) were purchased from Greatsolarcell. PbI_2_ was purchased from Alfa-Aesar. Poly(9,9-dioctyl-fluorene-co-N-(4-butylphenyl) diphenylamine (TFB) and poly(3,4-ethylenedioxythiophene)-poly(styrenesulfonate) (PEDOT:PSS) were purchased from Ossila. 2,2′,2′′-(1,3,5-Benzinetriyl)-tris(1-phenyl-1-H-benzimidazole) (TPBi) was purchased from Lumtec. Other materials for device fabrication were all purchased from Sigma-Aldrich.

### NIR PeLED fabrication

Indium tin oxide (ITO) glass substrates were sonicated in deionized water and ethanol for 15 min. The clean substrates were then treated by ultraviolet-ozone for 10 min. The ZnO nanocrystal (NCs) solution was spin-coated onto the substrate at 4,000 rpm for 30 s. After that, ZnO-NCs deposited substrates were transferred into a N_2_ filled glovebox and a layer of PEIE was deposited at 5000 rpm (0.05 wt%, in isopropanol) for 30 s, followed by annealing at 100 °C for 10 min. After cooling down to room temperature, the 3D perovskite layer (FAI: PbI_2_: ODEA::2:1:0.3 molar ratio in DMF solvent) was deposited at 3000 rpm for 30 s and annealed at 100 °C for 10 min. After that, TFB layer (12 mg ml^−1^) was deposited at 3000 rpm for 30 s. Finally, MoO_3_ (7 nm)/Au (80 nm) electrode were deposited under the base pressure of ∼2 × 10^−6^ torr. The device area is 7.25 mm^2^.

### Quasi-2D PeLED and OLED fabrication

Indium tin oxide (ITO) glass substrates were sonicated in deionized water and ethanol for 15 min. The clean substrates were then treated by ultraviolet-ozone for 20 min. The PEDOT:PSS solution was spin-coated onto the substrates at 4000 rpm for 40 s and annealed at 150 °C for 10 min. After annealing, substrates were transferred into a N_2_ filled glovebox. For quasi-2D PeLED, a layer of TFB (4 mg ml^−1^, in CB solvent) was deposited on top of PEDOT:PSS deposited substrate at 6000 rpm for 30 s, followed by annealing at 130 °C for 10 min. After cooling down to room temperature, the PVK (4 mg ml^−1^, in CB solvent) layer was deposited onto TFB deposited substrate at a spin-coating speed of 2000 rpm for 30 s, followed by annealing at 130 °C for 10 min. After cooling down to room temperature, the quasi-2D perovskite layer (POEA: MAI: CsI: PbI_2_:: 2: 2: 1.2: 2 molar ratio, in DMF solvent) was deposited at 4000 rpm for 30 s and annealed at 80 °C for 10 min. For OLED, TFB layer (10 mg ml^-1^, in CB solvent) was deposited at 3000 rpm for 60 s onto PEDOT:PSS deposited substrate, followed by annealing at 130 °C for 10 min. After that, 35 nm TPBi layer was deposited using thermal evaporation. Finally, LiF (1 nm)/Al (100 nm) electrode were deposited under the base pressure of ∼2 × 10^−6^ torr. The device area is 7.25 mm^2^.

### Steady-state characterisation of the PeLEDs

PeLED J–V–L and stability measurements were carried out at room temperature in a N_2_ filled glovebox. A Keithley 2400 source metre and a fibre integration sphere (FOIS-1) coupled with a QE Pro spectrometer (Ocean Optics) were used for the measurements. The PeLED devices are tested on top of the integration sphere and only forward light emission can be collected, consistent with the standard organic light-emitting diodes (OLED) characterisation method. The absolute PeLED emission was calibrated by a standard Visible–NIR light source (HL-3P-INT-CAL plus, Ocean Optics). The devices were swept from zero bias to forward bias. The time evolution of the electroluminescence external quantum efficiency (EQE_EL_) was measured using the same testing system.

### TrEL characterisation of the PeLEDs

All Tr. J and TrEL measurements carried out in a N_2_ filled glovebox. TrEL measurement was performed in forward direction as a function of frequency and pulse width. Supplementary Fig. 2 shows TrEL measurement setup, which was used to perform pulse operation on various types of device. A function generator (FG) (AFG3022C) was used to generate the periodic voltage pulse widths (from 10 µs to 90 ms) in the range of 10 Hz to 20 kHz; rise time and fall time are 9 ns, and the voltage height is 1.7 V. A fast Si photodetector (PD) with rise time (*t*_r_ = 1.5 ns) two orders of magnitude less than the RC time constant (≈0.13 µs) of the setup is used to detect the TrEL signal, which was recorded using a digital oscilloscope (DPO4104B-L). A 50 Ω resistance is connected in series with the PeLED, and across this resistance, the transient voltage is measured using an oscilloscope at 50 Ω termination. We calculated the transient current density using $\frac{{{{{{\rm{Transient}}}}}}\,{{{{{\rm{voltage}}}}}}}{{{{{{\rm{Resistance}}}}}}\times {{{{{\rm{Device}}}}}}\,{{{{{\rm{area}}}}}}}$ formula.

## Supplementary information

Supplementary Information

## Data Availability

The data that support the plots within this paper and other findings of this study are available from the corresponding authors upon reasonable request.

## Code availability

The code that supports the findings of this study is available from the corresponding authors upon reasonable request
