# Supplementary material for: Mobile ions determine the luminescence yield of perovskite light-emitting diodes under pulsed operation
Source: Nat Commun. 2021 Aug 12;12:4899. doi: 10.1038/s41467-021-25016-5 (PMC8361013; doi:10.1038/s41467-021-25016-5)
Supplement: Supplementary file 1 — Supplementary Information [file 41467_2021_25016_MOESM1_ESM.pdf]

Supplementary Information

Mobile ions determine the luminescence yield of perovskite light-emitting diodes under pulsed operation

Naresh Kumar Kumawat[1], Wolfgang Tress[2]*, Feng Gao[1]*

[1]Department of Physics, Chemistry and Biology (IFM), Linköping University, Linköping 58183, Sweden

[2]Institute of Computational Physics, Zurich University of Applied Sciences, Wildbachstr. 21, 8401 Winterthur, Switzerland

*Wolfgang Tress (wolfgang.tress@zhaw.ch)

*Feng Gao (feng.gao@liu.se)

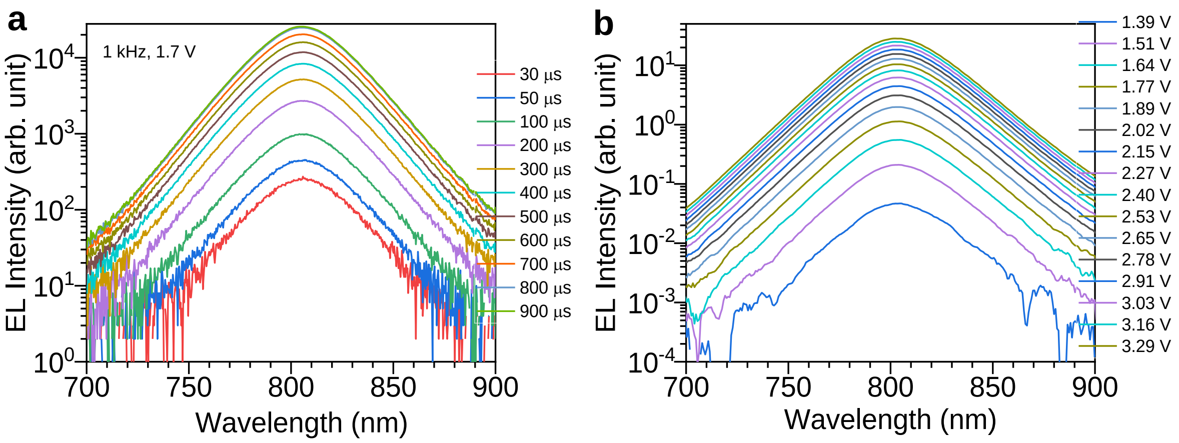

Supplementary Fig. 1 DC bias and pulse width dependent EL spectra of the NIR PeLED. (a) Semi-log EL spectra as function of electrical pulses at 1 kHz and 1.7 V. **(b)** Semi-log EL spectra as a function of DC bias. EL peak and full width at half maxima (fwhm) remain constant as a function of pulse width and DC bias, suggesting that recombination zones remains at the same position.

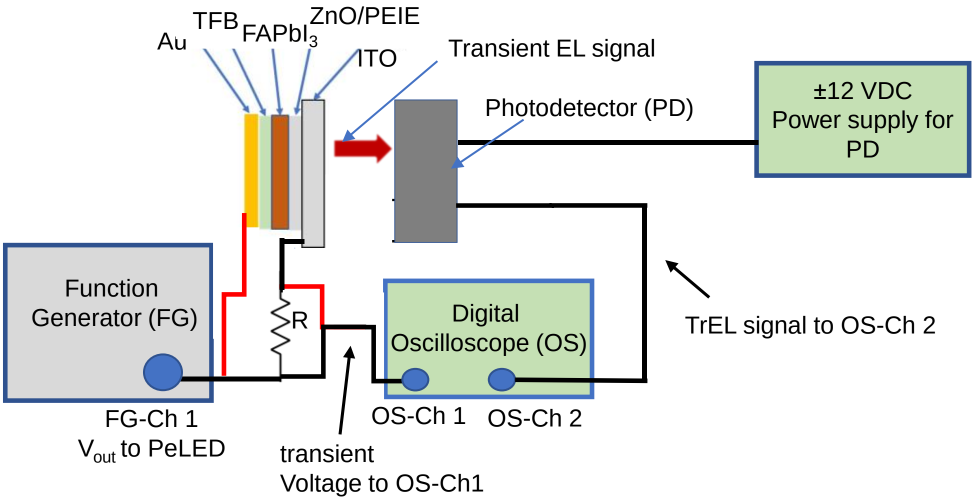

Supplementary Fig. 2 Transient electroluminescence (TrEL) measurement setup. A schematic diagram of the transient EL (TrEL) setup. A function generator (FG) was used to drive the device using various periodic voltage pulse widths and frequencies. Si photodetector (PD) output which is connected to a digital oscilloscope is used to detect the TrEL signal. R is resistance which is used to record transient voltage signal. The transient voltage was used to determine transient current density using $\frac{Transient\ voltage}{R\ X\ area}$ formula.

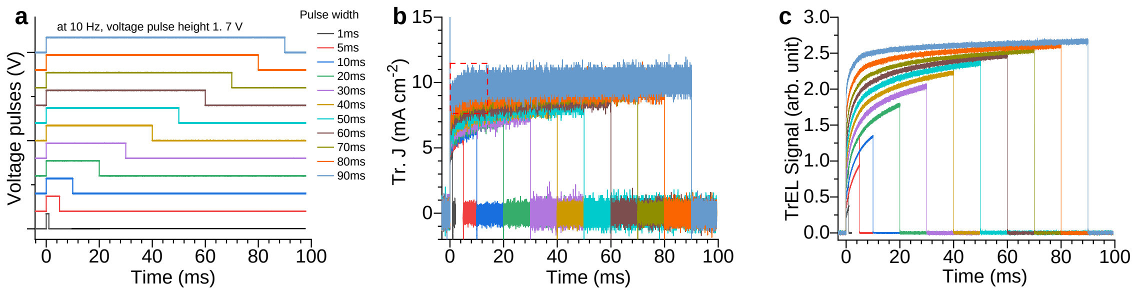

Supplementary Fig. 3 Pulse width dependent TrEL of the NIR PeLED at 10 Hz and 1.7 V. (a) Schematic diagram of the periodic voltage pulse widths (from 1 ms to 90 ms), which are used to excite the NIR PeLED. **(b)** Transient current density (Tr. J) and **(c)** TrEL signal of the NIR PeLED as a function of the pulse widths. Tr. J and TrEL intensity increase with an increasing pulse width. Also, Tr. J shows transient effect which is shown in supplementary Fig. 3b with red dash box.

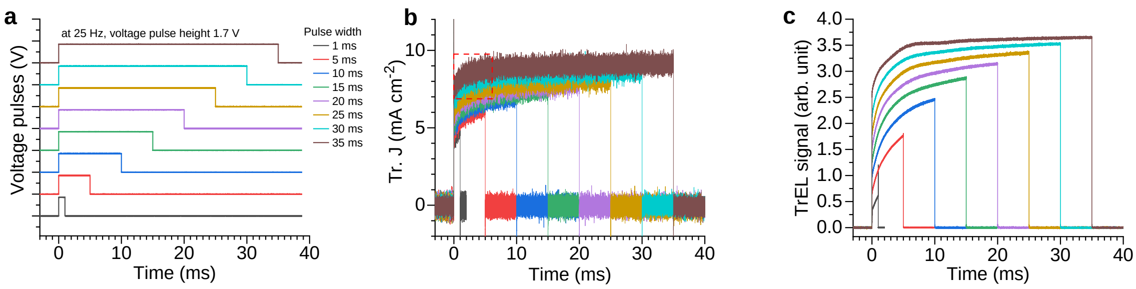

Supplementary Fig. 4 Pulse width dependent TrEL of the NIR PeLED at 25 Hz and 1.7 V. (a) Schematic diagram of the periodic voltage pulse widths (from 1 ms to 35 ms), which are used to excite the PeLED. **(b)** Transient current density (Tr. J) and **(c)** TrEL signal of the PeLED as a function of the pulse widths. Tr. J and TrEL intensity increase with an increasing pulse width. Also, Tr. J shows transient effect which is shown in supplementary Fig. 4b with red dash box.

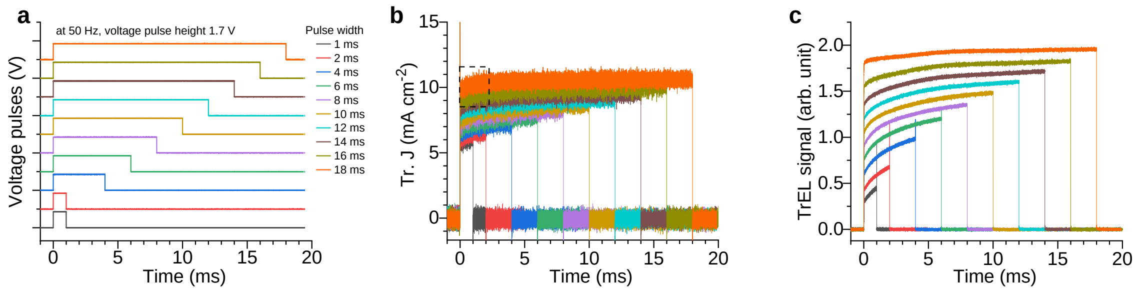

Supplementary Fig. 5 Pulse width dependent TrEL of the NIR PeLED at 50 Hz and 1.7 V. (a) Schematic diagram of the periodic voltage pulse widths (from 1 ms to 18 ms), which are used to excite the PeLED. **(b)** Transient current density (Tr. J) and **(c)** TrEL signal of the PeLED as a function of the pulse widths. Tr. J and TrEL intensity increase with an increasing pulse width. Also, Tr. J shows transient effect which is shown in supplementary Fig. 5b with black dash box.

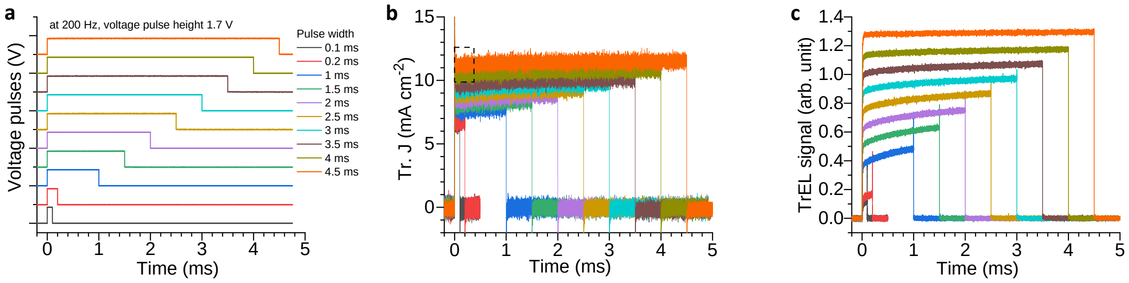

Supplementary Fig. 6 Pulse width dependent TrEL of the NIR PeLED at 200 Hz and 1.7 V. (a) Schematic diagram of the periodic voltage pulse widths (from 0.1 ms to 4.5 ms), which are used to excite the PeLED. **(b)** Transient current density (Tr. J) and **(c)** TrEL signal of the PeLED as a function of the pulse widths. Tr. J and TrEL intensity increase with an increasing pulse width. Also, Tr. J shows transient effect which is shown in supplementary Fig. 6b with black dash box.

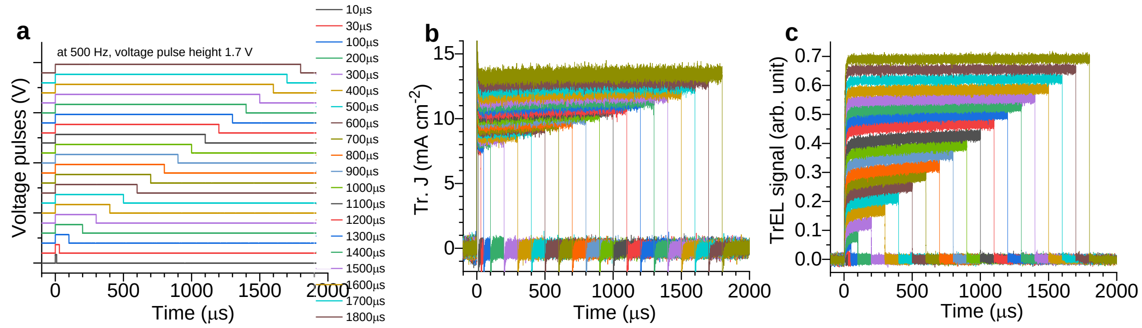

Supplementary Fig. 7 Pulse width dependent TrEL of the NIR PeLED at 500 Hz and 1.7 V. (a) Schematic diagram of the periodic voltage pulse widths (from 10 μs to 1800 μs), which are used to excite the PeLED. **(b)** Transient current density (Tr. J) and **(c)** TrEL signal of the PeLED as a function of the pulse widths. Tr. J and TrEL intensity increase with an increasing pulse width.

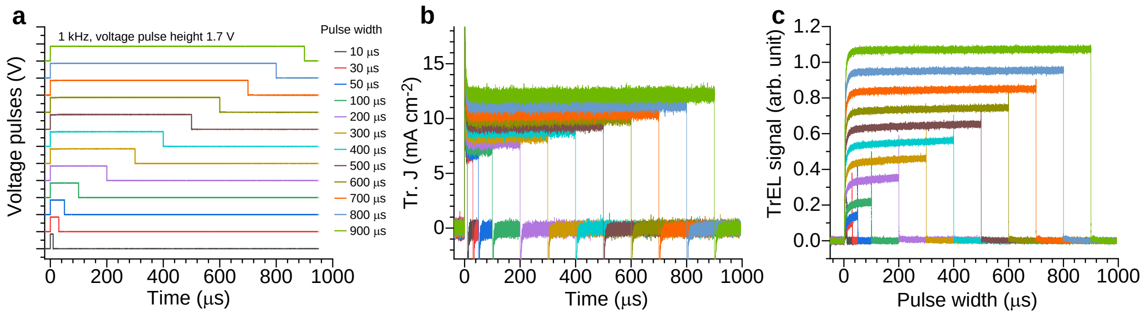

Supplementary Fig. 8 Pulse width dependent TrEL of the NIR PeLED at 1 kHz and 1.7 V. (a) Schematic diagram of the periodic voltage pulse widths (from 10 μs to 900 μs), which are used to excite the PeLED device. **(b)** Transient current density (Tr. J) and **(c)** TrEL signal of the PeLED as a function of the pulse widths. Tr. J and TrEL intensity increase with an increasing pulse width.

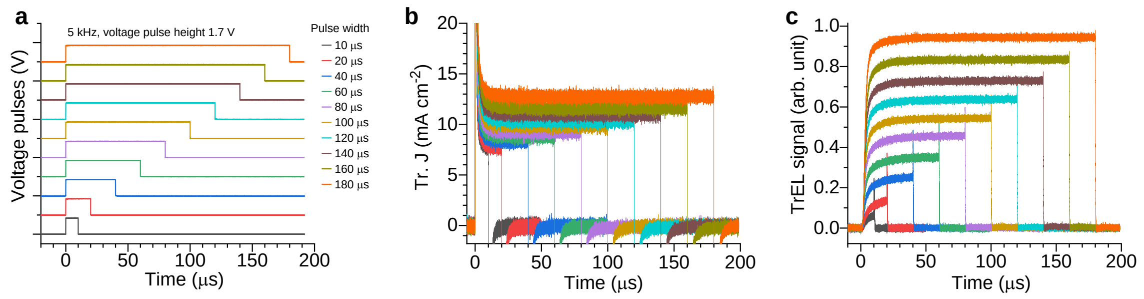

Supplementary Fig. 9 Pulse width dependent TrEL of the NIR PeLED at 5 kHz and 1.7 V. (a) Schematic diagram of the periodic voltage pulse widths (from 10 μs to 180 μs), which are used to excite the PeLED. **(b)** Transient current density (Tr. J) and **(c)** TrEL signal of the PeLED as a function of the pulse widths. Tr. J and TrEL intensity increase with an increasing pulse width.

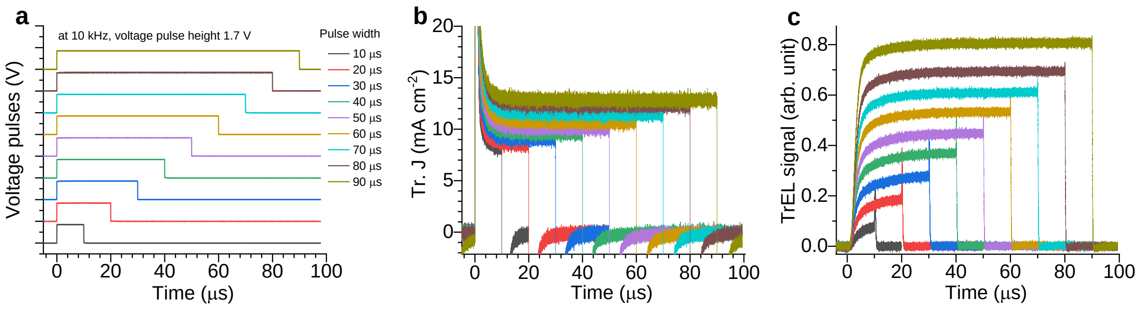

Supplementary Fig. 10 Pulse width dependent TrEL of the NIR PeLED at 10 kHz and 1.7 V. (a) Schematic diagram of the periodic voltage pulse widths (from 10 μs to 90 μs), which are used to excite the PeLED. **(b)** Transient current density (Tr. J) and **(c)** TrEL signal of the PeLED as a function of the pulse widths. Tr. J and TrEL intensity increase with an increasing pulse width.

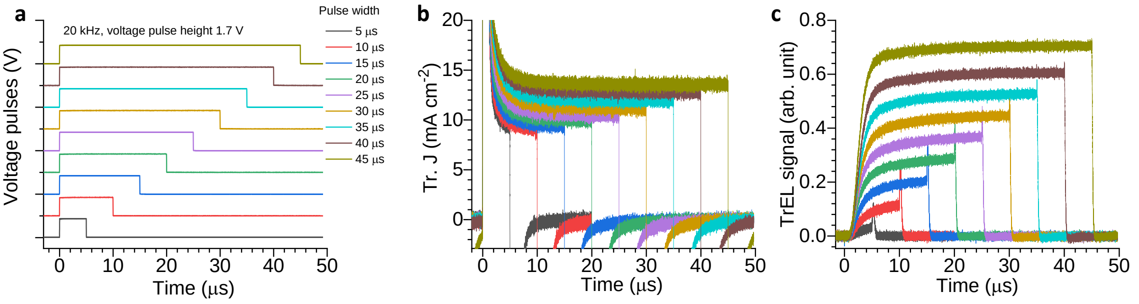

Supplementary Fig. 11 Pulse width dependent TrEL of the NIR PeLED at 20 kHz and 1.7 V. (a) Schematic diagram of the periodic voltage pulse widths (from 5 μs to 45 μs), which are used to excite the PeLED. **(b)** Transient current density (Tr. J) and **(c)** TrEL signal of the PeLED as a function of the pulse widths. Tr. J and TrEL intensity increase with an increasing pulse width.

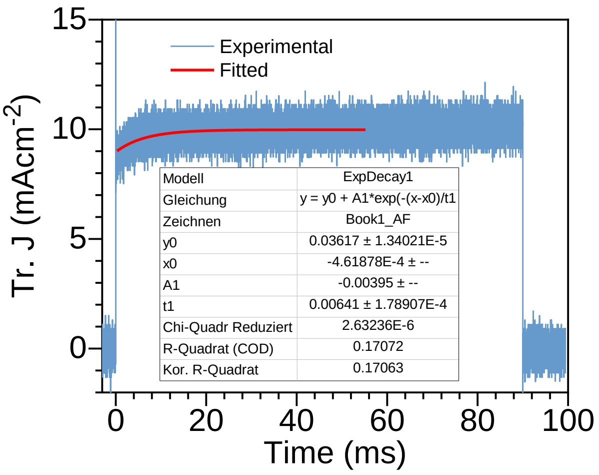

Supplementary Fig. 12 Transient current density of the NIR PeLED at 10 Hz and 1.7 V and ionic response. Transient current density (Tr. J) corresponds to 90 ms periodic voltage pulse width. Black and red curves are experimental and fitted data, respectively to determine the ionic response time of 6.4 ms.

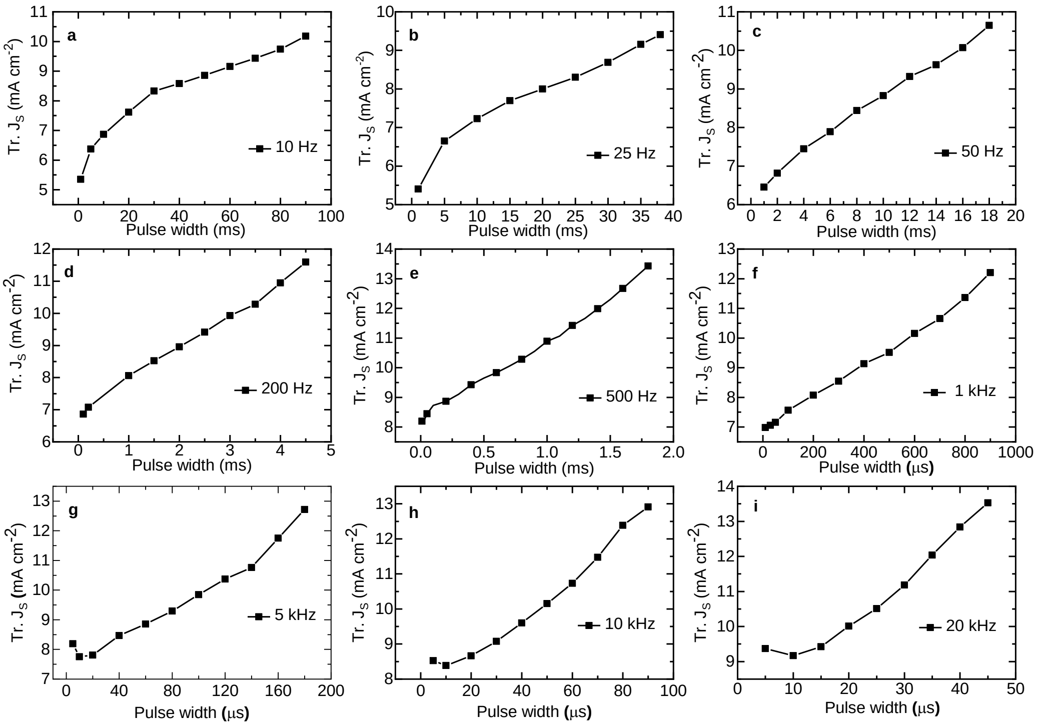

Supplementary Fig. 13 Pulse width dependent saturated transient current density (Tr. J_S) at 1.7 V. (a-i) Saturated transient current density (Tr. J_S) versus pulse widths for different frequencies. The variations in J_S are relatively constant (≈ 40%) from the small to long pulse width in the range of 10 Hz to 20 kHz.

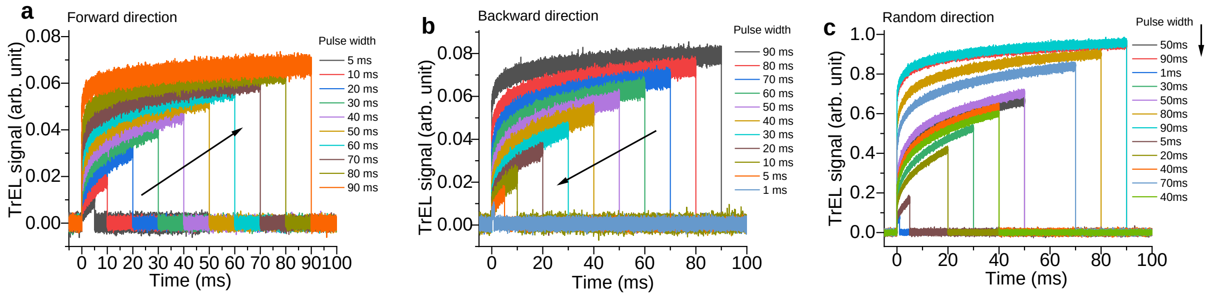

Supplementary Fig. 14 TrEL signal as a function of pulse width at 10 Hz and 1.7 V. (a) Forward direction. **(b)** Backward direction. **(c)** Random. Arrows show the measurement direction as a function of pulse widths. Forward, backward and random pulse width dependent TrEL data show that the TrEL is independent of the sequence at 10 Hz.

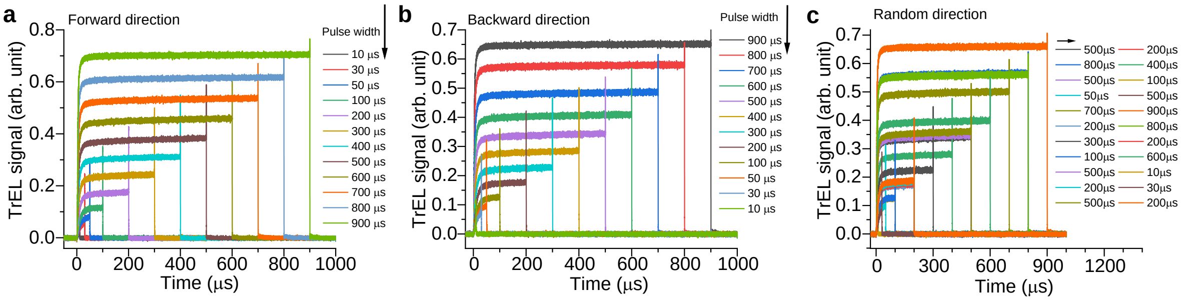

Supplementary Fig. 15 TrEL signal as a function of pulse width at 1 kHz and 1.7 V. (a) Forward direction. **(b)** Backward direction. **(c)** Random. Arrows show the measurement direction as a function of pulse widths. Forward, backward and random pulse width dependent TrEL data show that the TrEL is independent of the sequence at 1 kHz.

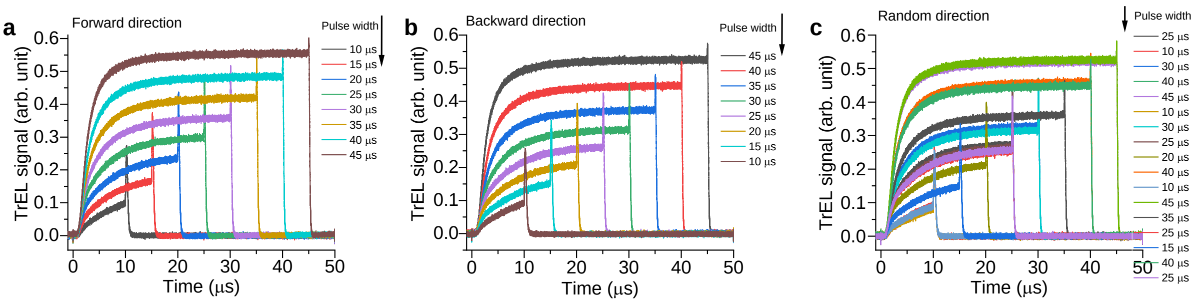

Supplementary Fig. 16 TrEL signal as a function of pulse width at 20 kHz and 1.7 V. (a) Forward direction. **(b)** Backward direction. **(c)** Random direction. Arrows show the measurement direction as a function of pulse widths. Forward, backward and random pulse width dependent TrEL data show that the TrEL is independent of the sequence at 20 kHz.

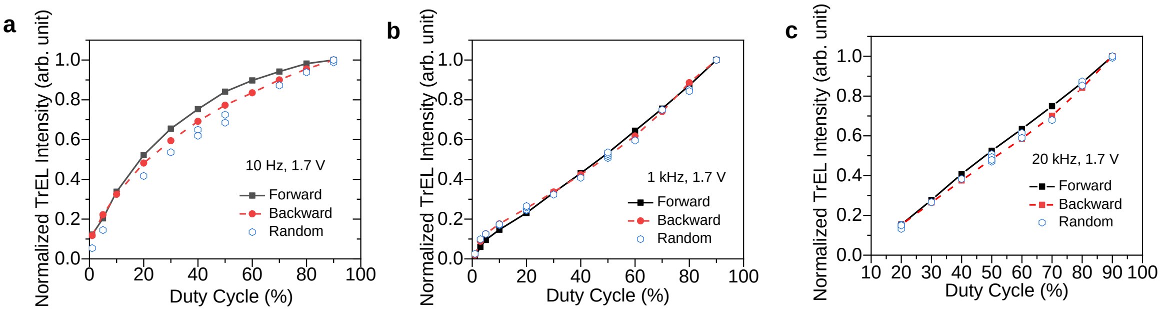

Supplementary Fig. 17 TrEL intensity versus duty cycle as function of frequency for forward, backward, and random direction. Normalized TrEL intensity ($TrEL_P$) versus duty cycle **(a)** 10 Hz. **(b)** 1 kHz. **(c)** 20 kHz. For these figures, we use plateau values ($TrEL_p$ intensity values at the end of the TrEL pulse) from TrEL data which are shown in Supplementary Fig. 14, Supplementary Fig. 15, and Supplementary Fig. 16. Measurement direction does not influence the trend of $TrEL_p$ characteristics.

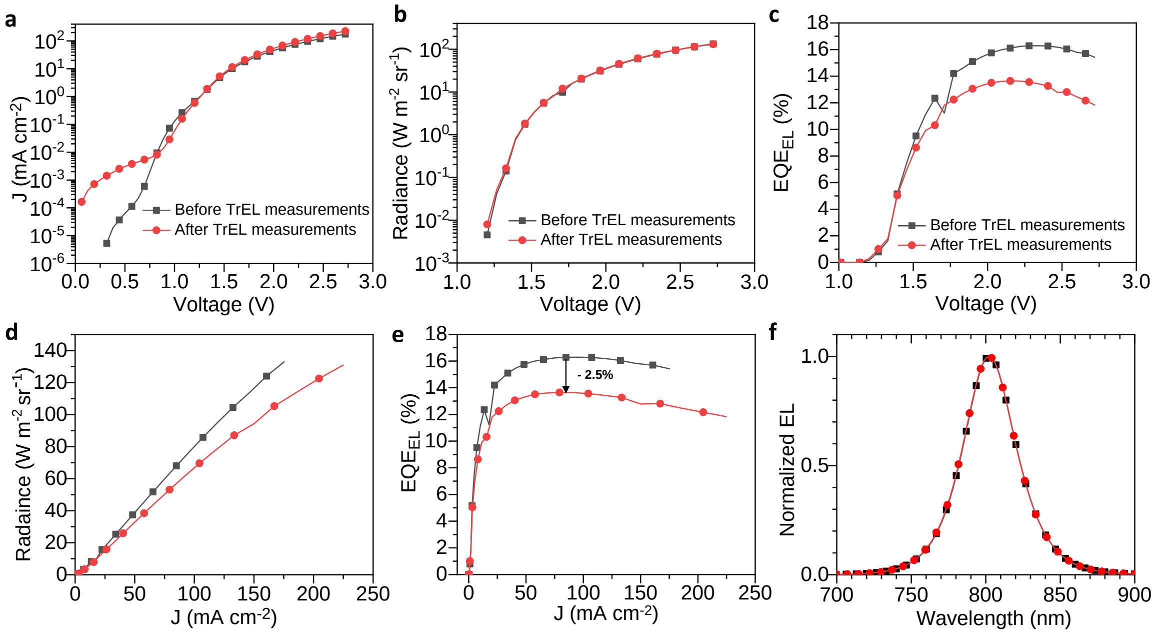

Supplementary Fig. 18 J-V-L characterizations before/after TrEL measurements of the NIR PeLED. (**a**) Current density, **(b)** Radiance and **(c)** EQE_{EL} versus voltage. **(d)** Radiance versus current density. **(e)** EQE_{EL} versus current density. **(f)** Normalized EL spectrums. After the TrEL measurements, device EQE_{EL} reduced from 16.2% to 13.6% (Supplementary Fig. 18e, with the black arrow), indicating that device shows similar level of performance after the TrEL measurements.

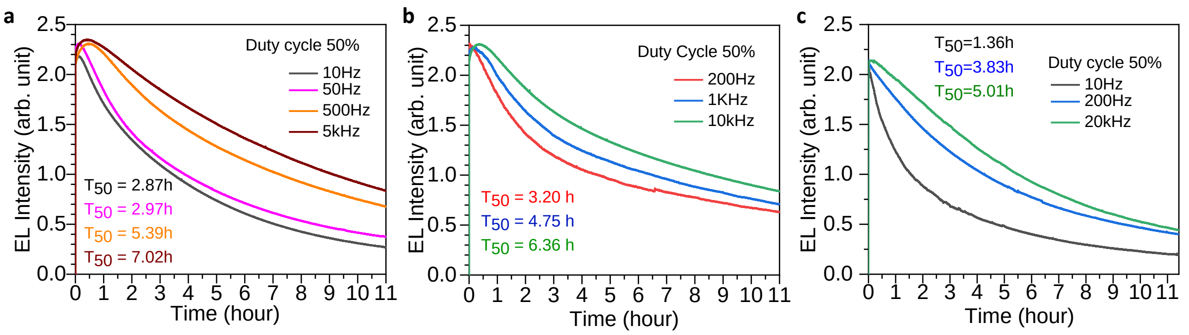

Supplementary Fig. 19 Frequency dependent stability curve of the NIR-PeLED at 50% duty cycle. (a) 10 Hz, 50 Hz, 500 Hz, and 5 kHz. **(b)** 200 Hz, 1 kHz, and 10 kHz. **(c)** 10 Hz, 200 Hz, and 20 kHz. Low frequency shows shorter lifetime as compared to high frequency. We have four pixels on each device. Therefore, in order to cover a wide range of frequencies, we have to use three devices. The stability curve for each frequency was measured on a different pixel. We note that half lifetime shows slight difference between devices.

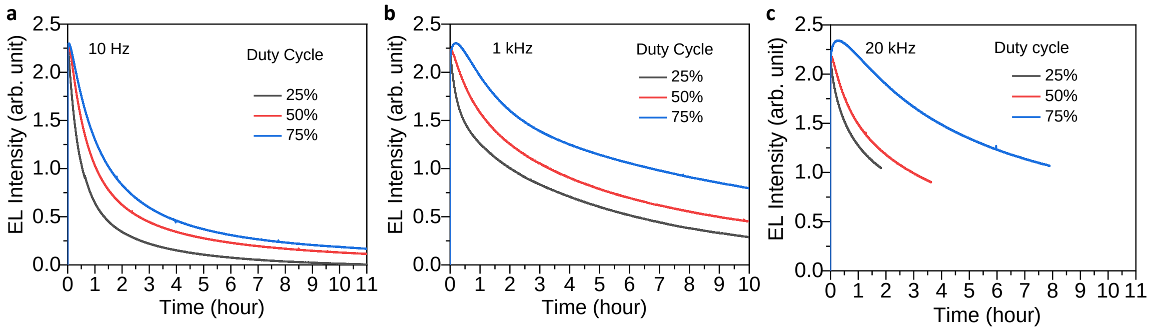

Supplementary Fig. 20 Pulse width (or duty cycle) dependent stability curve of the NIR-PeLED. (a) Stability curve at 10 Hz for 25% (pulse width 25 ms, applied bias 2.65 V), 50% (pulse width 50 ms, applied bias 2.07 V), and 75% (pulse width 75 ms, applied bias 1.92 V) duty cycle. **(b)** Stability curve at 1 kHz for 25% (pulse width 250 µs, applied bias 2.37 V), 50% (pulse width 50 µs, applied bias 2 V), and 75% (pulse width 75 µs, applied bias 1.92V) duty cycle. **(c)** Stability curve at 20 kHz for 25% (pulse width 12.5 µs, applied bias 2.5 V), 50% (pulse width 25 µs, applied bias 2.02 V), and 75% (pulse width 37.5 µs, applied bias 1.82 V) duty cycle.

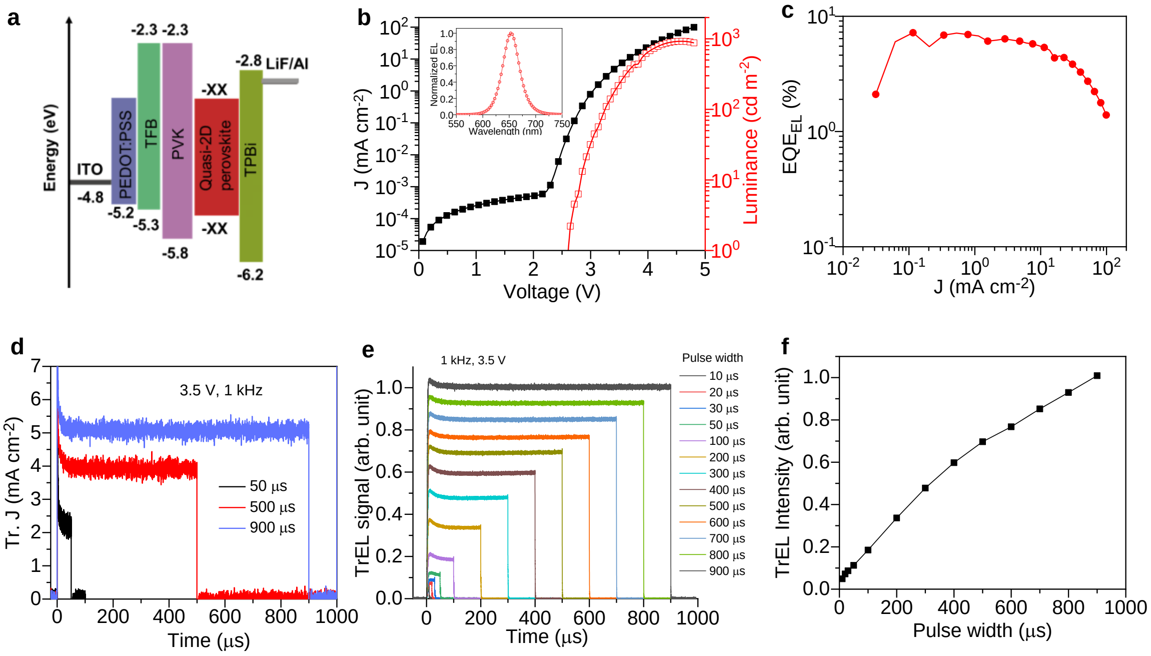

Supplementary Fig. 21. Device performance and TrEL Characteristics of the quasi-2D PeLED. **(a)** Energy level diagram of the quasi-2D PeLED. **(b)** J-V-L characterization of a quasi-2D PeLED, figure inset shows normalized EL spectrum that is recorded at 3.5 V, EL peak wavelength at 650 nm. **(c)** EQE versus current density. **(d)** Transient current density (Tr. J) as a function of voltage pulse widths (50 μs, 500 μs, and 900 μs) at 3.5 V and 1 kHz frequency. **(e)** TrEL signal as a function of the pulse widths. **(f)** TrEL intensity ($TrEL_p$) versus pulse widths.

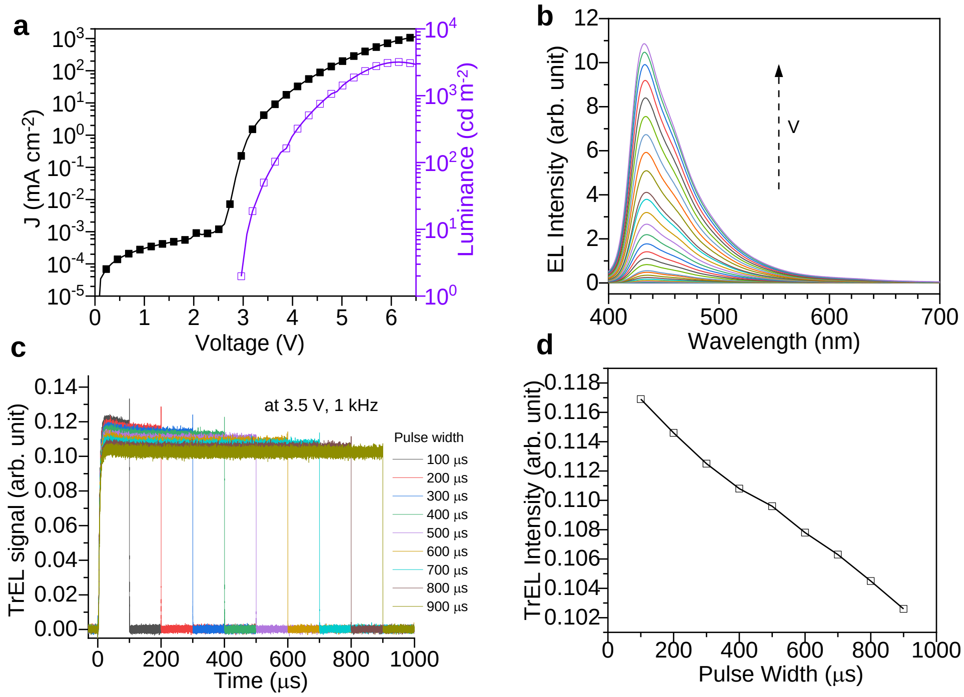

Supplementary Fig. 22 J-V-L and TrEL characteristics of the organic light-emitting diodes (OLED). (a) J-V-L characteristics of the OLED which is fabricated using ITO/PEDOT:PSS/TFB/TPBi/LiF/Al device structure, wherein TFB is emissive layer. **(b)** EL spectra as a function of bias, wherein the arrow shows increasing bias direction. **(c)** TrEL signal as function of the pulse widths at 1 kHz and 3.5 V. **(d)** TrEL intensity ($TrEL_p$) versus pulse width. OLED shows maximum luminance 3000 cd m^{-2} at 432 nm EL wavelength (Supplementary Fig.22a-b). This device is not efficient, but brightness level is quite high. We find that the OLED TrEL intensity decreases with an increasing pulse width (Supplementary Fig.22c-d). The change in the TrEL intensity is quite small as compared to PeLEDs (Fig. 2c, Fig. 2f and Supplementary Fig. 21e-f). Similar studies have been reported for other OLEDs, where small changes in TrEL intensity with an increasing pulse width were observed and overshoots were dependent on the device structure.[1, 2, 3]

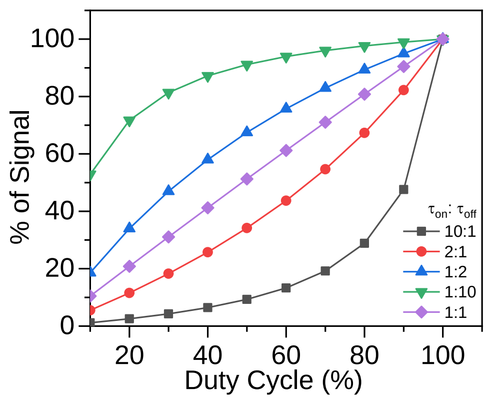

Supplementary Fig. 23 Plateau values of modelled TrEL as a function of duty cycle in the case of different time constants during on and off-time. The linear dependence on the duty cycle is lost and higher signals are achieved for a slower response during the off-time.

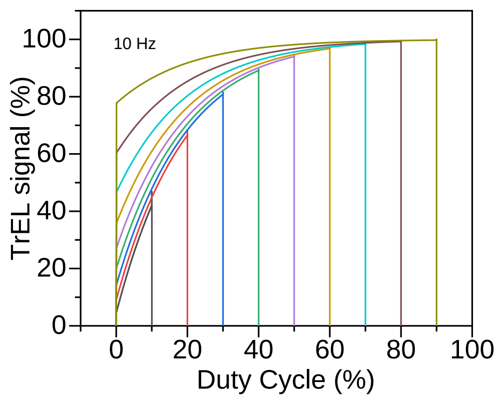

Supplementary Fig. 24 Modelled TrEL at 10 Hz as a function of duty cycle in the case of different time constants during on (20 µs) and off-time (40 µs). The slightly higher time constant in the off time might explain the different rise times seen in experiment.

Supplementary References

1. Cheon, K. O. & Shinar, J. Electroluminescence spikes, turn-off dynamics, and charge traps in organic light-emitting devices. *Phys. Rev. B* **69**, 201306 (2004).

2. Nikitenko, V. R. *et al.* The overshoot effect in transient electroluminescence from organic bilayer light emitting diodes: Experiment and theory. *Journal of Applied Physics* **81**, 7514–7525 (1997).

3. Pommerehne, J., Nikolaenkov, D. V., Nikitenko, V. R. & Bässler, H. Overshoot effect in transient electroluminescence from single layer organic light-emitting diodes. *Journal of Applied Physics* **90**, 5554–5560 (2001).
